# EarlyTect BCD Plus: A urine‐based dual site PENK methylation test for risk‐based cystoscopy triage in haematuria

**DOI:** 10.1002/bco2.70178

**Published:** 2026-03-06

**Authors:** Tae Jeong Oh, Bo‐Ram Bang, Jae Hee Hwang, Yangyei Seo, Seoyong Kim, Sujin Gu, Safedin Beqaj, Theo deVos, Justin Junguek Lee, Jin Zhong, Joseph D. Shirk, Katelyn W. Ke, John Vallone, Sungwhan An

**Affiliations:** ^1^ Genomictree, Inc. Daejeon Republic of Korea; ^2^ Promis Diagnostics, Inc. Irvine California USA; ^3^ Department of Medicine University of California, Riverside Riverside California USA; ^4^ Pathology & Laboratory Medicine Service VA Greater Los Angeles Medical Center Los Angeles California USA; ^5^ Department of Urology VA Greater Los Angeles Medical Center Los Angeles California USA; ^6^ Department of Urology David Geffen School of Medicine at UCLA Los Angeles California USA

**Keywords:** biomarkers, haematuria, molecular diagnostic techniques, proenkephalin, tumour, urinary bladder neoplasms

## Abstract

**Objectives:**

This study aims to develop and clinically evaluate EarlyTect BCD Plus, a urine‐based assay measuring two methylation sites of the *PENK* gene, and to assess its diagnostic performance and clinical utility according to haematuria risk stratification.

**Materials and Methods:**

A dual‐target quantitative methylation‐specific PCR assay was optimized using the *PENK* gene and evaluated in 892 patients with haematuria from Korea and the United States who underwent cystoscopy and histopathology. Urine DNA was analysed for two *PENK* methylation markers, and test results were interpreted using a combined algorithm. Diagnostic accuracy was assessed, and clinical utility was further analysed for patients stratified by the 2025 AUA/SUFU haematuria guideline risk categories.

**Results:**

In the pooled cohort (gross haematuria, *n* = 509; microhaematuria, *n* = 366; unspecified haematuria, *n* = 17), EarlyTect BCD Plus achieved a sensitivity of 87.7% (95% CI, 81.5%–92.5%), specificity of 82.5% (95% CI, 79.6%–85.2%) and negative predictive value (NPV) of 97.0% (95% CI, 95.5%–98.0%). Sensitivity for Ta‐LG tumours improved to 60.5% compared with the original single‐marker assay, while high‐grade tumours were detected with 96.6% sensitivity. In the intermediate‐risk group, NPVs were 99.1% for all BC and 100% for high‐grade BC.

**Conclusions:**

EarlyTect BCD Plus significantly enhances detection of Ta‐LG bladder cancer while maintaining high specificity. Its high NPV supports use as a non‐invasive adjunct and triage tool, allowing deferral of cystoscopy in selected haematuria patients.

## 1 INTRODUCTION

Bladder cancer (BC) is the fifth most common malignancy worldwide, with approximately 550 000 new cases and 200 000 deaths annually.^1^ Haematuria is the most frequent presenting symptom, accounting for up to 20% of urological evaluations.^2^, ^3^ The incidence of BC ranges from 0.4% to 2.6% for microscopic haematuria to as high as 20% in gross haematuria.^4^, ^5^ Consequently, many patients without malignancy still undergo cystoscopy and imaging.^5^

Cystoscopy remains the standard practice for diagnosing BC during the initial evaluation of patients with haematuria. However, it is invasive, costly and often poorly tolerated and may fail to detect up to 30% of lesions, particularly flat or small tumours, including carcinoma in situ.^6^ As a non‐invasive adjunct, urine cytology provides high specificity but has limited sensitivity, detecting as few as 12% of low‐grade (LG) tumours.^7^

An important challenge in urological practice is to safely minimize unnecessary negative cystoscopies while ensuring timely cancer detection in patients at higher risk due to age, sex, smoking and carcinogen exposure. To address this, the American Urological Association (AUA) guidelines recommend stratifying patients with haematuria according to their risk factors for urothelial carcinoma (UC), an approach that reduces unnecessary diagnostic evaluation while maintaining effective UC detection. Within this framework, the updated 2025 AUA/SUFU microhaematuria guideline endorses the selective use of validated urine‐based tumour markers (UBTMs) in appropriately counselled intermediate‐risk patients. This highlights a paradigm shift in evaluation: Risk stratification defines which patients may benefit, and UBTMs provide a non‐invasive adjunct to help balance cancer detection with avoidance of unnecessary cystoscopies.^8^

Consequently, a sensitive, non‐invasive urine assay that can triage patients for cystoscopy represents an attractive solution. Several urinary biomarker–based assays, including NMP22, BTA stat and UroVysion, have received FDA approval and have been discussed in clinical guidelines. These assays demonstrate overall sensitivities of 58%–78% and specificities of 74%–88%, yet their sensitivity for Ta‐LG tumours remains low (30%–48%).^9^, ^10^, ^11^ In addition, recently developed in vitro diagnostic assays such as EarlyTect BCD, Cxbladder, AssureMDx and Xpert have been evaluated, incorporated into clinical discussions and recently included in the AUA/SUFU guideline for microhaematuria evaluation.^8^ These assays underscore the growing role of urinary biomarkers in refining risk‐adapted evaluation of haematuria while highlighting the continuing need for improved sensitivity, particularly for early‐stage and low‐grade disease.

DNA methylation represents an attractive biomarker strategy because of its early and stable occurrence during tumourigenesis, and several methylation assays have shown promising diagnostic value across cancers.^12^, ^13^, ^14^, ^15^ Among these, hypermethylation of the proenkephalin (*PENK*) gene was identified and developed by our group as a promising urinary biomarker for BC detection. In a recent multicentre prospective study, we validated the single‐target methylation assay (EarlyTect BCD), which demonstrated 78.1% sensitivity and 88.8% specificity in patients with haematuria.^16^, ^17^ The assay showed high sensitivity for high‐grade and higher‐stage tumours, but its performance for Ta‐LG disease was suboptimal, with sensitivity as low as 32.6%.

To overcome this limitation, we developed an improved dual‐target assay, EarlyTect BCD Plus (BCD Plus), by incorporating an additional methylation site to complement the original biomarker. This study aimed to validate the diagnostic performance of BCD Plus in patients with haematuria and compare it with the original single‐target assay. Furthermore, we evaluated its clinical potential as a non‐invasive triage tool aligned with the 2025 AUA/SUFU risk‐based guideline, providing context relative to established urinary biomarkers such as Cxbladder and other approved assays.

## 2 MATERIALS AND METHODS

### 2.1 Design

This study used remnant samples previously collected in multicentre prospective trials of patients with haematuria, including 790 samples from Korea and 88 samples from the United States, together with an additional 14 samples from patients prospectively enrolled in the United States between March 2022 and June 2024.^16^, ^18^ In total, 892 urine samples obtained from the two cohorts were tested using BCD Plus. All laboratory personnel conducting the methylation assays were blinded to clinical and pathological information. In the previous studies, ethical approval was obtained from the institutional review board, and all participants provided written informed consent.

### 2.2 Procedures

Urine samples were preserved in the Urine Collection Kit (Genomictree, Inc., Republic of Korea). DNA was isolated using the GT NUCLEIC ACID PREP Kit (Genomictree, Inc.) and subjected to bisulfite conversion using the EZ DNA Methylation‐Gold Kit (Zymo Research, CA, USA). Converted DNA was eluted in 20 μL of elution buffer and used immediately for LTE‐qMSP.

The BCD Plus assay was performed once for each sample. Quantification of methylation was performed using the ΔCT method, where ΔCT = CT (*PENK*) − CT (*COL2A1*), and methylation levels were expressed as 35 − ΔCT.^16^, ^18^, ^19^ Higher values of 35 − ΔCT represented higher levels of methylation. For samples in which *PENK* was undetectable, the 35 − ΔCT value was set to 20. Reactions were considered valid if the *COL2A1* CT was <33.0. Cut‐off values were prespecified at >32.0 for Marker‐1^16^, ^17^ and determined at >32.0 for Marker‐2 from this study (Table S1). For BCD Plus assay, a sample was interpreted as positive if either Marker‐1 or Marker‐2 exceeded its respective cut‐off and negative only if both fell below threshold.

### 2.3 Statistical analysis

All statistical analyses were performed using MedCalc (v20.115; MedCalc Software Ltd.). The cut‐off value for Marker‐1 was prespecified at 32.0 based on prior clinical studies.^16^, ^18^ For Marker‐2, the optimal cut‐off value was determined using receiver operating characteristic (ROC) curve analysis. In addition to conventional approaches (e.g., maximizing the Youden index), we applied the method proposed by Ünal in which the cut‐off is selected to minimize the sum of absolute differences between sensitivity/specificity and the overall diagnostic performance (AUC) (Table S1).^20^ Test results were dichotomized as methylation‐positive (1) or methylation‐negative (0) for statistical analysis.

Diagnostic performance was assessed using sensitivity, specificity, positive predictive value (PPV), negative predictive value (NPV) and AUC, each reported with corresponding 95% confidence intervals (CIs). Pairwise comparisons between Marker‐1 and BCD Plus were conducted using McNemar's test for paired binary outcomes. Positive and negative likelihood ratios were calculated for the BCD Plus test. All statistical tests were two‐sided unless otherwise specified, and *p* values < 0.05 were considered statistically significant.

## 3 RESULTS

### 3.1 Patients

The combined analysis included a total of 892 patients presenting with haematuria, consisting of 155 patients diagnosed with BC and 737 patients without BC malignancy (Table 1). In the Korean cohort, 151 patients were diagnosed with BC, while 639 had no evidence of BC (no‐BC). In the US cohort, four patients were diagnosed with BC, while 98 patients had no evidence of BC. The BC group was predominantly male (83.9%) and older (mean age 69.0), compared to the no‐BC group (54.5% male, mean age 65.2). Gross haematuria was more frequently observed in the BC group (82.6%) compared to the no‐BC group (51.7%). Conversely, microscopic haematuria was more prevalent in the no‐BC group (46.0% vs. 17.4%). A history of smoking was reported more often in the BC group (56.7%) than in the no‐BC group (28.4%). Among the no‐BC group, three patients were diagnosed with upper tract urothelial carcinoma (UTUC, ureter cancer and renal pelvic cancer) (data not shown).

**TABLE 1:** Demographic and clinical characteristics of study cohorts.

Characteristics	Korean cohort, *n* (%)		US cohort, *n* (%)		Pooled cohort, *n* (%)	
	BC (*n* = 151)	No‐BC^a^ (*n* = 639)	BC (*n* = 4)	No‐BC^a^ (*n* = 98)	BC (*n* = 155)	No‐BC^a^ (*n* = 737)
Sex, no. (%)						
Male	126 (83.4)	305 (47.7)	4 (100)	97 (99.0)	130 (83.9)	402 (54.5)
Female	25 (16.6)	334 (52.3)	‐	1 (1.0)	25 (16.1)	335 (45.5)
Age, mean (range)	68.8 (42 to 91)	64.2 (40 to 94)	76.3 (64 to 89)	72.0 (31 to 96)	69.0 (42 to 91)	65.2 (31 to 96)
Haematuria, no. (%)						
Microscopic	27 (17.9)	310 (48.5)	‐	29 (29.6)	27 (17.4)	339 (46.0)
Gross	124 (82.1)	329 (51.5)	4 (100)	52 (53.1)	128 (82.6)	381 (51.7)
Unknown	‐	‐	‐	17 (17.3)	‐	17 (2.3)
Smoking history, no. (%)						
Never	63 (41.7)	429 (67.1)	‐	‐	63 (40.6)	429 (58.2)
Former	52 (34.4)	133 (20.8)	‐	3 (3)	52 (33.5)	136 (18.5)
Current	36 (23.8)	77 (12.1)	‐	‐	36 (23.2)	77 (10.4)
Unknown	‐	‐	4 (100)	95 (97)	4 (2.6)	95 (12.9)
Tumour stage, no. (%)						
Ta	66 (43.7)		3 (75.0)		69 (44.5)	
CIS	2 (1.3)		‐		2 (1.3)	
T1	62 (41.1)		1 (25.0)		63 (40.6)	
T2	16 (10.6)		‐		16 (10.3)	
T3	5 (3.3)		‐		5 (3.2)	
Tumour grade, no. (%)						
Low	37 (24.5)		1 (25.0)		38 (24.5)	
High	114 (75.5)		3 (75.0)		117 (75.5)	

Abbreviations: BC, bladder cancer; No‐BC, no bladder cancer.

^a^ No‐BC includes patients with benign causes of haematuria such as ureter stone, benign prostate hyperplasia, inactive urinary tract inflammation, use of anticoagulants and others.

### 3.2 Performance of BCD Plus for haematuria

As outlined in Table 2, in the pooled cohort, BCD Plus assay had a sensitivity of 87.7% (136/155; 95% CI, 81.5%–92.5%) and a specificity of 82.5% (608/737; 95% CI, 79.6%–85.2%), with an AUC of 0.851 (95% CI, 0.826–0.874). The three UTUC patients that were positive for BCD Plus in this study were included as no‐BC, slightly lowering the specificity. By comparison, Marker‐1 (the original EarlyTect BCD test) showed a sensitivity of 78.1% (121/155) and specificity of 88.6% (653/737), while Marker‐2 demonstrated a sensitivity of 86.5% (134/155) and specificity of 87.4% (644/737), with corresponding AUCs of 0.833 and 0.869, respectively (Tables 2 and S2 and Figure S1A–C).

**TABLE 2:** Comparison of diagnostic performance between EarlyTect BCD and EarlyTect BCD Plus in the pooled cohort.

Parameter	Marker‐1	Marker‐2	BCD Plus	Marker‐1 vs. BCD Plus	
				Incremental difference	*p*‐value*
Sensitivity, % [95% CI]	78.1 [70.7–84.3]	86.5 [80.0–91.4]	87.7 [81.5–92.5]	+9.6	<0.0001
Ta (*n* = 69)	50.7 [38.4–63.0]	71.0 [58.8–81.3]	71.0 [58.8–81.3]	+20.3	<0.0001
Ta‐LG (*n* = 38)	31.6 [17.5–48.7]	57.9 [40.8–73.7]	60.5 [43.4–76.0]	+28.9	<0.001
Ta‐HG (*n* = 31)	74.2 [55.4–88.1]	87.1 [70.2–96.4]	87.1 [70.2–96.4]	+12.9	0.125
CIS (*n* = 2)	100 [15.8–100]	100 [15.8–100]	100 [15.8–100]	NA	NA
T1 (*n* = 63)	100 [94.3–100]	100 [94.3–100]	100 [94.3–100]	NA	NA
T2, T3 (*n* = 21)	100 [83.9–100]	100 [83.9–100]	100 [83.9–100]	NA	NA
High‐grade disease^a^ (*n* = 117)	93.2 [87.0–97.0]	95.7 [90.3–98.6]	96.6 [91.5–99.1]	+3.4	0.125
Specificity, % [95% CI]	88.6 [86.1–90.8]	87.4 [84.8–89.7]	82.5 [79.6–85.2]	−6.1	<0.0001
PPV, % [95% CI]	59.0 [53.7–64.2]	59.0 [54.1–63.8]	51.3 [47.1–55.5]	−7.7	NA
NPV, % [95% CI]	95.1 [93.4–96.3]	96.8 [95.4–97.9]	97.0 [95.5–98.0]	+1.9	NA

Abbreviations: CI, confidence interval; NPV, negative‐predictive value.

^a^ High‐grade disease indicates Ta‐HG, CIS, T1, T2 and T3.

* *p* values were calculated using McNemar's test for sensitivity and specificity.

Stage‐specific analyses revealed that BCD Plus assay detected 96.6% of Ta high‐grade (Ta‐HG) and higher stages tumours (113/117; 95% CI, 91.5%–99.1%). For early‐stage disease, the sensitivity for Ta tumours overall was 71.0% (49/69), with Ta‐LG detected at 60.5% (23/38). These results represent substantial improvements compared with Marker‐1 alone (Ta: 50.7%; Ta‐LG: 31.6%) and modest gains over Marker‐2 alone (Ta: 71.0%; Ta‐LG: 57.9%). All carcinoma in situ (CIS; *n* = 2) were detected by all Marker‐1, Marker‐2 and the combination in BCD Plus (100% sensitivity) (Table 2).

Pairwise comparison confirmed these improvements. For BCD Plus, a statistically significant gain in sensitivity over Marker‐1 (overall incremental difference: +9.6%, *p* < 0.0001) was observed, with the largest incremental benefit observed in Ta‐LG tumours (incremental difference: +28.9%, *p* < 0.001) (Table 2).

Predictive values also showed improvement; the NPV was 95.1% for Marker‐1, 96.8% for Marker‐2 and 97.0% (95% CI, 95.5%–98.0%) for BCD Plus. The PPV was 51.3% (95% CI, 47.1%–55.5%). Performance was consistent across sex, age, haematuria type, smoking status and tumour stage, with significant associations observed with tumour grade (*p* < 0.001; Table S3).

### 3.3 Haematuria risk stratification‐based performance

In addition to the overall diagnostic performance, we further assessed the potential utility of BCD Plus as a triage tool by analysis of patients stratified by the AUA/SUFU haematuria risk criteria.^7^ This analysis was restricted to the Korean cohort (Tables 3 and S4) due to a lack of data on smoking history and RBC counts in the US cohort. The details of smoking history in the Korean cohort were limited, so we adopted the modified risk classification as shown in Table S5. In the low‐risk cohort, no cases of bladder cancer (BC) were observed, and all 21 BCD Plus‐negative (BCDP–) patients were true negatives (Figure S2), indicating that unnecessary cystoscopies may be safely avoided in this population. Among intermediate‐risk patients, only one Ta‐LG case among the three BC cases was BCDP–, resulting in a high NPV of 99.1% (Table 3). In the high‐risk microhaematuria cohort, BCD Plus demonstrated strong performance, correctly identifying 91.7% (22/24) of cancers, with only two Ta‐LG cases missed. Similarly, in patients with gross haematuria, BCD Plus detected 87.1% (108/124) of cancer cases, with the majority of missed cases limited to low‐grade tumours (12 Ta‐LG and 4 Ta‐HG). These findings underscore that the test reliably detects clinically significant cancers while reducing invasive evaluations among non‐cancer cases (Table S6). The rule‐out rate was 76.6% for all microhaematuria patients and 83.6% for intermediate‐risk patients (Table 3).

**TABLE 3:** Performance of EarlyTect BCD Plus in patients with microhaematuria stratified by risk categories.

Parameter	Low risk (*n* = 26)	Intermediate risk (*n* = 134)	High risk (*n* = 177)	Total (*n* = 337)
Sensitivity, % [95% CI]				
Overall BC	‐	66.7 [9.4–99.2]	91.7 [73.0–99.0]	88.9 [70.8–97.7]
High‐grade disease	‐	100 [15.8–100]	100 [82.4–100]	100 [83.9–100]
Specificity, % [95% CI]	80.8 [60.7–93.5]	84.7 [77.4–90.4]	80.4 [73.2–86.4]	82.3 [77.5–86.4]
PPV, % [95% CI]				
Overall BC	‐	9.1 [3.9–19.7]	42.3 [34.2–50.8]	30.4 [24.9–36.5]
High‐grade disease	‐	9.1 [6.3–13.0]	38.8 [31.5–46.6]	28.0 [23.4–33.1]
NPV, % [95% CI]				
Overall BC	100 [84.6–100]	99.1 [96.7–99.8]	98.4 [94.2–99.6]	98.8 [96.7–99.6]
High‐grade disease	100 [84.6–100]	100 [96.7–100]	100 [97.1–100]	100 [98.6–100]
LR+, % [95% CI]	‐	4.4 [1.8–10.7]	4.7 [3.3–6.6]	5.0 [3.8–6.6]
LR−, % [95% CI]	‐	0.4 [0.1–2.0]	0.1 [0.0– 0.4]	0.1 [0.1–0.4]
Rule‐out rate, % [95% CI]	80.8 [60.6–93.4]	83.6 [76.4–88.9]	70.6 [63.3–77.2]	76.6 [72.2–80.5]

Abbreviations: CI, confidence interval; LR+, positive likelihood ratio; LR−, negative likelihood ratio; NPV, negative‐predictive value; PPV, positive‐predictive value.

### 3.4 Analysis of likelihood ratios (LRs)

In the microhaematuria cohort, for BCD Plus negative patients, LR− was 0.1 (95% CI, 0.1–0.4). For BCD Plus positive patients, LR+ was 5.0 (95% CI, 3.8–6.6) (Table S7).

## 4 DISCUSSION

In this study, we determined the performance of the enhanced EarlyTect BCD Plus, a dual‐target methylation assay designed to improve sensitivity of bladder cancer detection in patients presenting with haematuria. The original EarlyTect BCD test demonstrated strong performance for high‐grade and advanced‐stage tumours but limited sensitivity for Ta‐LG disease, a limitation shared by many existing urine‐based assays.^16^, ^18^ By targeting two CpG‐rich regions of the *PENK* gene, the BCD Plus test demonstrated markedly improved diagnostic performance. Sensitivity for Ta‐LG tumours nearly doubled, from 31.6% with the single‐site assay to 60.5%, while overall sensitivity increased from 78.1% to 87.7% and the specificity remained stable at 82.5%. The NPV also improved, from 98.2% to 98.8% in the microhaematuria cohort and this improvement aligns with the AUA/SUFU guideline's emphasis on achieving a high NPV for UBTMs. These findings indicate that integrating methylation signals from multiple regions within a single gene can enhance assay sensitivity while maintaining clinically acceptable specificity.

The BCD Plus also outperformed traditional urine‐based tests such as cytology and NMP22 (Table S8) in this study. While both cytology and NMP22 maintained high specificity, sensitivity for bladder cancer, including high‐grade disease, was considerably lower. In contrast, BCD Plus achieved higher sensitivity while maintaining acceptable specificity, suggesting greater reliability as a non‐invasive adjunct for haematuria evaluation.

The AUA/SUFU guideline for microhaematuria evaluation recognizes a number of UBTMs for evaluation of intermediate risk haematuria patients primarily based on NPV. Among these, Cxbladder Triage was evaluated as having an A level of evidence, supported by the prospective multicentre STRATA trial.^7^ In that randomized study comparing Cxbladder Triage with cystoscopy in patients with microhaematuria, use of the test significantly reduced unnecessary cystoscopies while maintaining diagnostic safety for bladder cancer detection. The study reported an overall NPV of 98.6% at 7.8% prevalence, 100% at 1.3% prevalence in lower‐risk patients and 97.6% at 10.3% prevalence in higher‐risk patients. In comparison, BCD Plus achieved an overall NPV of 98.8% at 8.0% prevalence, with 100% in low‐risk (0% prevalence), 99.1% in intermediate‐risk (2.2% prevalence), and 98.4% in high‐risk (13.6% prevalence) patients. Notably, BCD Plus achieved an NPV exceeding 99.0% in intermediate‐risk patients, comparable to the performance of CxBladder in the lower‐risk group, which corresponds to the intermediate‐risk category in current guidelines. In high‐risk patients, BCD Plus also maintained a robust NPV despite being assessed in a higher‐prevalence cohort, supporting its reliability for rule‐out use across all risk groups. PPVs also followed the expected prevalence gradient, rising from 9.1% in intermediate‐risk patients to 42.3% in high‐risk and 62.8% in gross haematuria. These findings underscore the stable diagnostic behaviour of BCD Plus across clinical subgroups, providing reassurance from negative results and additional value from positive ones.

Overall, BCD Plus achieved an NPV of 97.0% for all bladder cancers and 99.4% for high‐grade disease, consistent with the observed prevalence of BC in the study cohort (17.4% overall and 13.7% high‐grade). Risk‐stratified analyses confirmed consistent performance.

Current AUA/SUFU guidelines do not recommend urinary biomarkers for use in high‐risk microhaematuria patients or those with gross haematuria.^8^ However, the relatively high PPV and specificity of BCD Plus suggest that it may offer added benefit by identifying patients who are most likely to have BC. In real‐world practice, adherence to cystoscopy remains suboptimal. A prior study reported that 87% of high‐risk patients with microscopic haematuria did not undergo guideline‐recommended evaluation, with only 12.8% referred for cystoscopy and 10.4% receiving cytology.^21^ Such underutilization contributes to delayed diagnosis, with approximately one quarter of bladder cancers still detected at advanced stages. While referral rates are somewhat higher in gross haematuria, adherence remains incomplete. In this context, the high PPV of BCD Plus can help identify those patients most likely to have cancer, supporting timely cystoscopy and improving compliance with guideline‐based evaluation.

While NPV and PPV are informative indicators, both are influenced by disease prevalence. LRs complement these metrics by providing prevalence‐independent insight into diagnostic strength.^22^, ^23^ Incorporation of BCD Plus substantially enhanced diagnostic performance, yielding an LR+ greater than 5—representing moderately strong rule‐in evidence—and an LR– of 0.1, indicating strong rule‐out capability. In the microhaematuria cohort with an estimated pre‐test probability of approximately 8%, these likelihood ratios translated into a post‐test probability of bladder cancer below 1% after a negative result and about 30% after a positive result (Table S7). The markedly low post‐test probability following a negative result suggests that bladder cancer could be effectively ruled out in most patients. In the study, the potential cystoscopy rule‐out rate for the overall population was 76.9%, increasing to >80% in the low and intermediate risk groups. In contrast, the marked increase in post‐test probability following a positive result provides strong evidence to prioritize further diagnostic evaluation, supporting its role in ruling in disease. These post‐test probabilities highlight the discriminatory capacity of BCD Plus to enhance risk stratification for patients with microhaematuria and to refine clinical decision‐making regarding cystoscopy.

Beyond bladder cancer, BCD Plus detected all UTUC cases (3 of 3) that were missed by cystoscopy at initial evaluation. This is consistent with prior findings showing detection of all UTUC cases (9 of 9) by the original BCD test.^24^ Given the shared urothelial origin of bladder and upper tract tumours, *PENK* methylation may serve as a common molecular signature of malignant transformation. Although exploratory, these results suggest possible applicability across the urinary tract, which warrants further validation in larger prospective studies.

Taken together, these data suggest a potential dual clinical role for BCD Plus across all haematuria risk categories. Consistently high NPVs and a low LR– demonstrate reliable exclusion of disease, supporting deferral of cystoscopy when appropriate. Conversely, relatively high PPVs in high‐risk and gross haematuria groups and an increased LR+ provide actionable evidence for prioritizing cystoscopy in those most likely to harbour malignancy.

This study has several limitations. It used remnant specimens from prospective cohorts, and the Korean cohort was relatively larger than the United States cohort, which may limit generalizability. The United States cohort was not included in the risk stratification analysis, and smoking history was not recorded in sufficient detail to enable full guideline‐based classification. The number of cancers in some subgroups, particularly the intermediate‐risk cohort, was limited, constraining precision of those estimates. The exploratory UTUC findings were based on a very small number of cases and should be interpreted cautiously. Additionally, the current analysis was observational and not a randomized controlled study. Nevertheless, the inclusion of 892 patients across two independent cohorts provides a robust dataset enhancing the reliability of these findings. The larger sample size, including a greater number of Ta‐LG cases, also allowed for a more reliable assessment of test performance compared with earlier studies, which were limited by smaller cohorts.

## 5 CONCLUSIONS

In conclusion, EarlyTect BCD Plus was developed to address the sensitivity limitations of the original single‐target assay. The dual‐target test substantially improves detection of Ta‐LG and early‐stage bladder cancer while maintaining acceptable specificity. When evaluated in the context of AUA/SUFU risk stratification guidelines and benchmarked against other guideline‐endorsed biomarkers such as Cxbladder, BCD Plus demonstrates strong potential as a non‐invasive adjunct that enhances both diagnostic accuracy and adherence to cystoscopy recommendations. If confirmed in prospective multicentre trials, BCD Plus could help reduce unnecessary cystoscopies, improve patient compliance, and optimize overall outcomes in haematuria management.

## AUTHOR CONTRIBUTIONS

*Conception, design and supervision:* Sungwhan An and Tae Jeong Oh. *Drafting the manuscript:* Tae Jeong Oh, Bo‐Ram Bang, Seoyong Kim, Justin Junguek Lee and Theo deVos. *Data analysis, interpretation and statistical analysis:* Tae Jeong Oh, Bo‐Ram Bang, Jin Zhong, Justin Junguek Lee, Theo deVos and Safedin Beqaj. *Lab testing and data acquisition:* Jae Hee Hwang, Yangyei Seo, Bo‐Ram Bang, Seoyong Kim and Sujin Gu. *Patient recruitment and data collection:* Jin Zhong, Joseph D. Shirk, Katelyn W. Ke and John Vallone. All authors participated in manuscript review and revision prior to submission.

## CONFLICT OF INTEREST STATEMENT

J.H. Hwang, Y. Seo, T.J. Oh and S. An are employees of Genomictree, Inc. B.‐R. Bang, S. Kim, J.J. Lee, S. Gu, S. Beqaj, T. deVos and S. An are employees of Promis Diagnostics, Inc. J.H. Hwang, Y. Seo, T.J. Oh, S. An, B.‐R. Bang, S. Kim and J.J. Lee are shareholders of Genomictree, Inc. J.J. Lee and S. An are shareholders of Promis Diagnostics, Inc. All other authors report no disclosures.

## Supporting information


**Figure S1.** *PENK* methylation levels and ROC curve analysis for detecting BC in hematuria patients using urine‐derived DNA. A. Methylation levels of Marker‐1 and Marker‐2 in urine samples from individuals with BC and no‐BC in the Korean cohort. Methylation levels are expressed as 35‐ΔCT values; ***P < 0.001 by Mann–Whitney test. B. Methylation levels of Marker‐1 and Marker‐2 in the US cohort, expressed as 35‐ΔCT values with cutoff lines indicated. **P < 0.01, ***P < 0.001 by Mann–Whitney test. C. ROC curves showing the performance of the *PENK* methylation test in discriminating BC from no‐BC samples in both the Korean and the US cohorts.
**Figure S2.** The subjects were grouped into low‐, intermediate (Int)‐, high‐risk, and gross hematuria (GH) categories based on risk factors such as sex, age, RBC count, and smoking history. The test results of the BCD Plus test (BCDP), categorized as negative (−) or positive (+), were compared with clinical diagnoses.
**Table S1.** Determination of the optimal cutoff value for Marker‐2 in discriminating bladder cancer from patients with hematuria.
**Table S2.** Results of EarlyTect BCD Plus according to clinical diagnosis.
**Table S3.** Association of clinicopathologic parameters with EarlyTect BCD Plus results in 155 patients with bladder cancer.
**Table S4.** Demographic characteristics of the Korean cohort for risk classification.
**Table S5.** Modified risk classification for patients with microhematuria in this study.
**Table S6.** Comparison of EarlyTect BCD Plus performance between microhematuria and gross hematuria.
**Table S7.** Pre‐test and post‐test probabilities of detecting BC in the pooled cohort with microhematuria.
**Table S8.** Performance comparison of BCD Plus, Cytology, and NMP22.
