# Supplementary material for: EarlyTect BCD Plus: A urine‐based dual site PENK methylation test for risk‐based cystoscopy triage in haematuria
Source: BJUI Compass. 2026 Mar 6;7(3):e70178. doi: 10.1002/bco2.70178 (PMC12966605; doi:10.1002/bco2.70178)
Supplement: Supplementary file 1 — Figure S1. PENK methylation levels and ROC curve analysis for detecting BC in hematuria patients using urine‐derived DNA. A. Methylation levels of Marker‐1 and Marker‐2 in urine samples from individuals with BC and no‐BC in the Korean cohort. Methylation levels are expressed as 35‐ΔCT values; ***P < 0.001 by Mann–Whitney test. B. Methylation levels of Marker‐1 and Marker‐2 in the US cohort, expressed as 35‐ΔCT values with cutoff lines indicated. **P < 0.01, ***P < 0.001 by Mann–Whitney test. C. ROC curves showing the performance of the PENK methylation test in discriminating BC from no‐BC samples in both the Korean and the US cohorts. Figure S2. The subjects were grouped into low‐, intermediate (Int)‐, high‐risk, and gross hematuria (GH) categories based on risk factors such as sex, age, RBC count, and smoking history. The test results of the BCD Plus test (BCDP), categorized as negative (−) or positive (+), were compared with clinical diagnoses. Table S1. Determination of the optimal cutoff value for Marker‐2 in discriminating bladder cancer from patients with hematuria. Table S2. Results of EarlyTect BCD Plus according to clinical diagnosis. Table S3. Association of clinicopathologic parameters with EarlyTect BCD Plus results in 155 patients with bladder cancer. Table S4. Demographic characteristics of the Korean cohort for risk classification. Table S5. Modified risk classification for patients with microhematuria in this study. Table S6. Comparison of EarlyTect BCD Plus performance between microhematuria and gross hematuria. Table S7. Pre‐test and post‐test probabilities of detecting BC in the pooled cohort with microhematuria. Table S8. Performance comparison of BCD Plus, Cytology, and NMP22. [file BCO2-7-e70178-s001.pdf]

Supplementary Results

Cutoff determination for Marker-2

To determine the optimal cutoff value of Marker-2 for distinguishing BC from nonmalignant hematuria, a ROC curve analysis was performed using 790 Korean samples. The cutoff value was selected at the point where the sensitivity and specificity most closely aligned with the AUC, minimizing the deviation between these metrics, as outlined by Ünal.[1]

Initial ROC analysis indicated a potential cutoff at 33.2. To refine this estimate, adjacent values (32.0, 32.5, 33.0, and 33.5) were further assessed, with recalculation of sensitivity, specificity, and AUC for each. A threshold of 32.0 was ultimately chosen, as it demonstrated the most balanced alignment of sensitivity, specificity, and AUC, thereby offering the most clinically meaningful value for the Marker-2 methylation assay (Supplemental Table S1).

Performance of BCD Plus for each cohort

In the Korean cohort, both Marker-1 and Marker-2 exhibited significantly elevated methylation levels across all stages of BC compared with no-BC controls, with particularly pronounced separation observed in Ta-HG and higher-stage tumors ($P < 0.001$, Mann–Whitney) (Supplementary Figure S1A). The dual-target BCD Plus assay achieved a sensitivity of 87.4% (132/151; 95% CI, 81.1–92.2%) and a specificity of 81.4% (520/639; 95% CI, 78.1–84.3%), resulting in an AUC of 0.844 (95% CI, 0.817–0.869).

Similarly, in the independent US cohort, both markers showed higher methylation in BC compared with no-BC controls (Marker-1, $P < 0.001$; Marker-2, $P < 0.01$), confirming the consistency of these findings across different populations (Supplementary Figure S1B). The assay achieved a

sensitivity of 100% (4/4; 95% CI, 39.8–100%) and a specificity of 89.8% (88/98; 95% CI, 82.0–95.0%), with an AUC of 0.949 (95% CI, 0.887–0.983).

Supplementary Figure Legend

Supplementary Figure S1. *PENK* methylation levels and ROC curve analysis for detecting BC in hematuria patients using urine-derived DNA. A. Methylation levels of Marker-1 and Marker-2 in urine samples from individuals with BC and no-BC in the Korean cohort. Methylation levels are expressed as 35-ΔCT values; ***P < 0.001 by Mann-Whitney test. B. Methylation levels of Marker-1 and Marker-2 in the US cohort, expressed as 35-ΔCT values with cutoff lines indicated. **P < 0.01, ***P < 0.001 by Mann-Whitney test. C. ROC curves showing the performance of the *PENK* methylation test in discriminating BC from no-BC samples in both the Korean and the US cohorts.

Supplementary Figure S2. The subjects were grouped into low-, intermediate (Int)-, high-risk, and gross hematuria (GH) categories based on risk factors such as sex, age, RBC count, and smoking history. The test results of the BCD Plus test (BCDP), categorized as negative (-) or positive (+), were compared with clinical diagnoses.

Supplementary Figure S1.

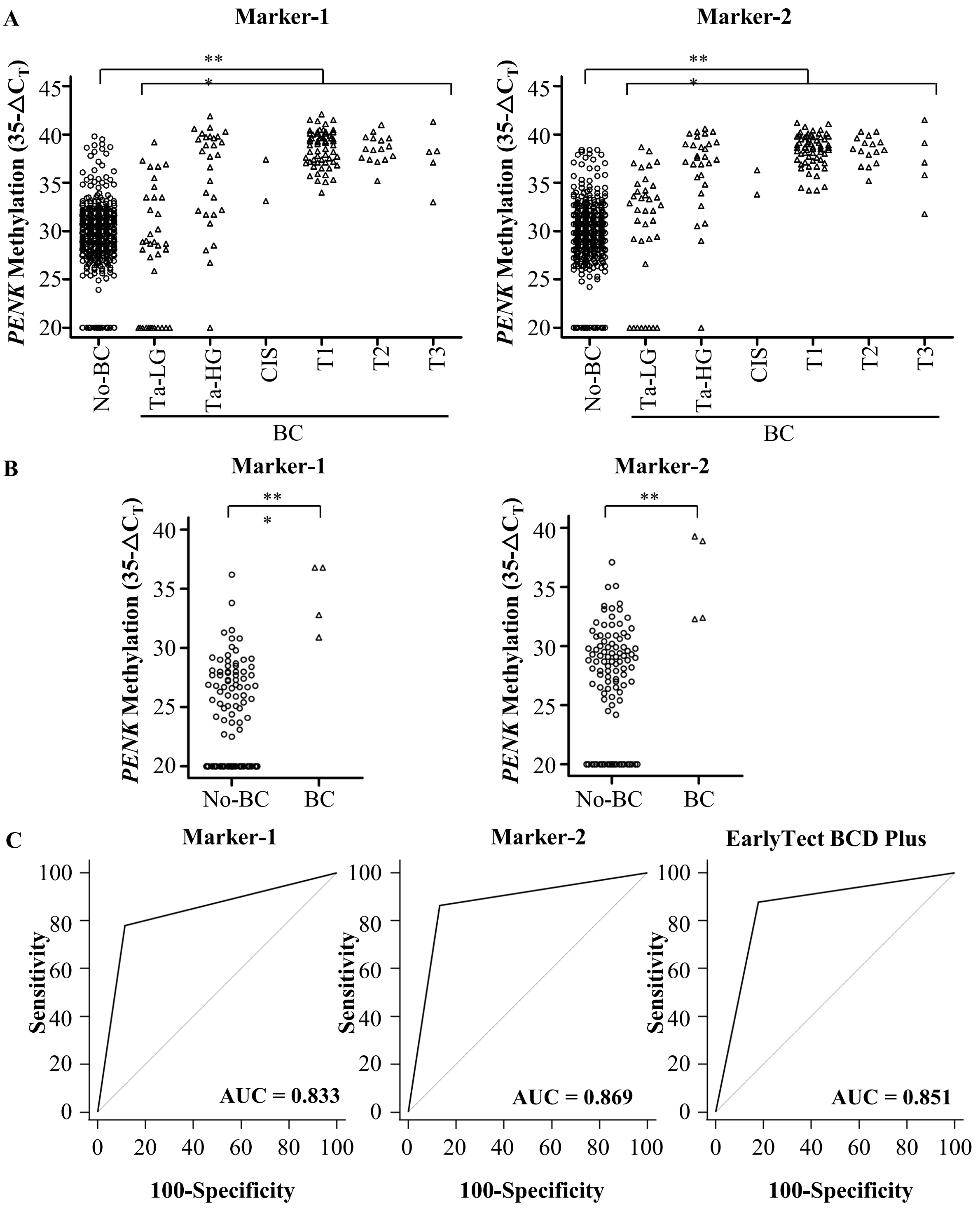

Supplementary Figure S2

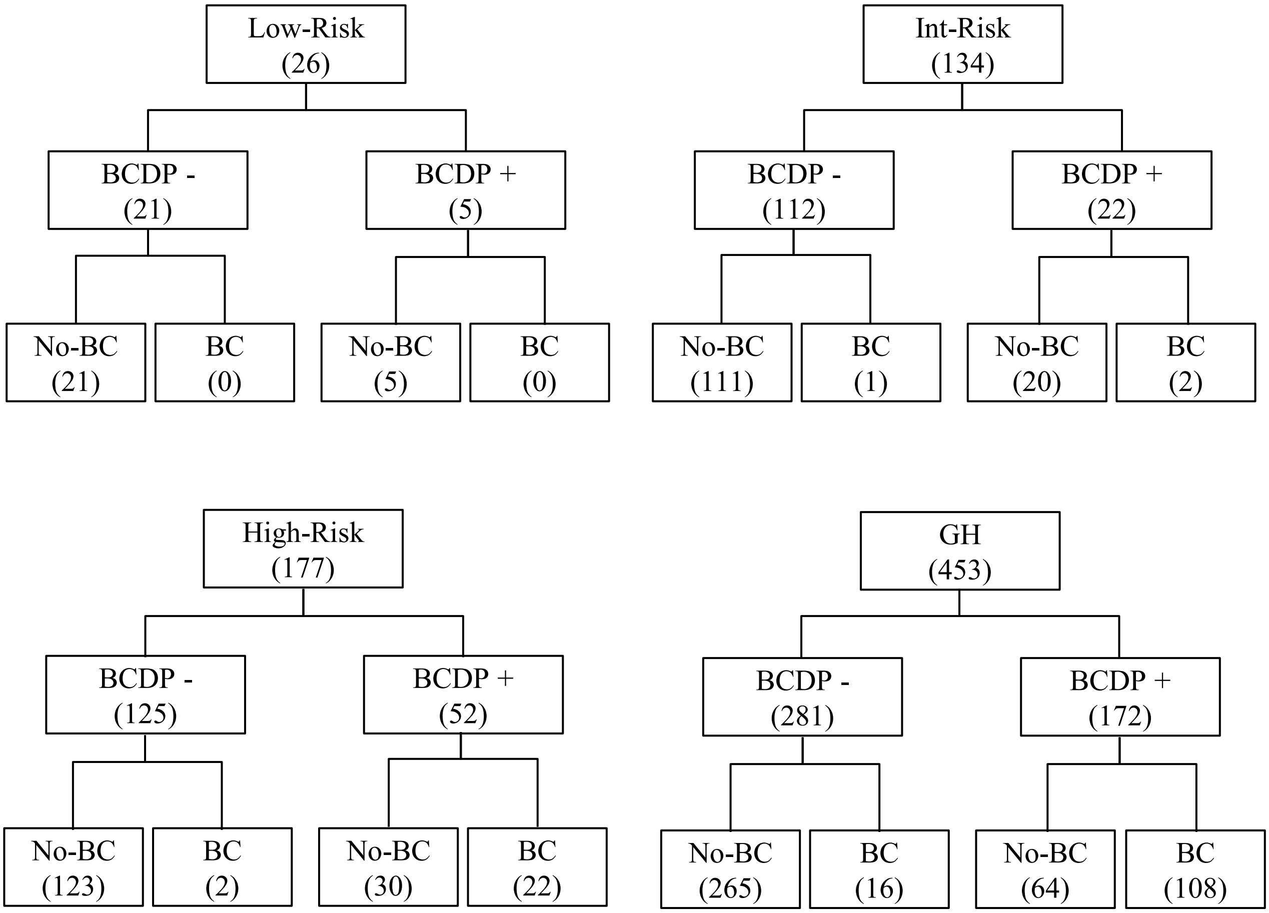

Supplementary Tables

Supplementary Table S1. Determination of the optimal cutoff value for Marker-2 in discriminating bladder cancer from patients with hematuria

Cutoff value	32.0	32.5	33.0	33.2	33.5
**|Sensitivity-AUC|[a]**	|0.861 – 0.866| = 0.005	|0.841 – 0.868| = 0.027	|0.821 – 0.876| = 0.055	|0.821 – 0.879| = 0.058	|0.801 – 0.871| = 0.070
**|Specificity-AUC|[a]**	|0.870 – 0.866| = 0.004	|0.895 – 0.868| = 0.027	|0.931 – 0.876| = 0.055	|0.937 – 0.879| = 0.058	|0.941 – 0.871| = 0.070
**Sum of values**	0.005 + 0.004 = 0.009	0.027 + 0.027 = 0.054	0.055 + 0.055 = 0.110	0.058 + 0.058 = 0.116	0.070 + 0.070 = 0.140

[a] Difference in absolute values

Abbreviation: AUC, Area Under Curve

Supplementary Table S2. Results of EarlyTect BCD Plus according to clinical diagnosis

	Marker-1		Marker-2		BCD Plus	
Test results	Negative	Positive	Negative	Positive	Negative	Positive
**Korean cohort (n = 790)**						
No Bladder Cancer (n = 639)	557	82	556	83	520	119
Bladder Cancer (n = 151)	33	118	21	130	19	132
**US cohort (n = 102)**						
No Bladder Cancer (n = 98)	96	2	88	10	88	10
Bladder Cancer (n = 4)	1	3	-	4	-	4
**Pooled cohort (n = 892)**						
No Bladder Cancer (n = 737)	653	84	644	93	608	129
Bladder Cancer (n = 155)	34	121	21	134	19	136

Supplementary Table S3. Association of clinicopathologic parameters with EarlyTect BCD Plus results in 155 patients with bladder cancer.

Parameter	Samples tested, *n*	*PENK* methylation positive, *n* (%)	*P* value*
**Sex**			
Male	130	115 (88.5)	0.513
Female	25	21 (84.0)	
**Age, years**			
< 60	29	25 (86.2)	0.758
≥60	126	111 (88.1)	
**Hematuria type**			
Microscopic	27	24 (88.9)	1.000
Gross	128	112 (87.5)	
**Smoking history**			
Never	63	54 (85.7)	
Former	52	47 (90.4)	0.256
Current	36	28 (77.8)	
**Tumor stage**			
Ta, CIS, T1	134	115 (85.8)	0.077
T2, T3	21	21 (100)	
**Tumor grade**			
Low	38	23 (60.5)	< 0.001
High	117	113 (96.6)	

**P* values were calculated using Fisher's exact test.

Supplementary Table S4. Demographic characteristics of the Korean cohort for risk classification

**Characteristics**	**Korean cohort (n = 790)**	
	Females (*n* = 359)	Males (*n* = 431)
**Age category, No. (%), y**	64 (41 to 94)	66 (40 to 91)
<40	-	-
40-59	116 (32.3)	113 (26.2)
≥60	243 (67.7)	318 (73.8)
**Smoking history, No. (%)**		
Never	348 (96.9)	144 (33.4)
Former	5 (1.4)	180 (41.8)
Current	6 (1.7)	107 (24.8)
**RBC count, No. (%)**		
3-10 RBC/HPF	89 (24.8)	73 (16.9)
11-25 RBC/HPF	56 (15.6)	24 (5.6)
≥ 25 RBC/HPF	214 (59.6)	334 (77.5)

Supplementary Table S5. Modified risk classification for patients with microhematuria in this study

**Risk**	**Low (All of the following)**	**Intermediate (One or more of the following)**	**High (One or more of the following)**
**Sex and Age**	• Women age <60<br>• Men age <40	• Women age ≥60<br>• Men age 40-59	• Women not categorized by age alone<br>• Men age ≥60
**Smoking History**	• Never smoker	• Former smoker	• Current smoker
**RBC count**	• 3-10 RBC/HPF	• 11-25 RBC/HPF	• ≥ 25 RBC/HPF

Supplementary Table S6. Comparison of EarlyTect BCD Plus performance between microhematuria and gross hematuria.

**Parameter**	**Microhematuria (n = 337)**	**Gross hematuria (n = 453)**	**All hematuria (n = 790)**
**Sensitivity,** % [95% CI]			
Overall BC	88.9 [70.8-97.7]	87.1 [80.0-92.4]	87.7 [81.5-92.5]
High-grade disease [a]	100 [83.9-100]	95.7 [89.4-98.8]	96.6 [91.5-99.1]
**Specificity,** % [95% CI]	82.3 [77.5-86.4]	80.6 [75.9-54.7]	82.5 [79.6-85.2]
**PPV,** % [95% CI]			
Overall BC	30.4 [24.9-36.5]	62.8 [55.1-70.0]	52.8 [46.4-59.1]
High-grade disease [a]	28.0 [23.4-33.1]	51.7 [44.0-59.4]	44.0 [37.8-50.4]
**NPV,** % [95% CI]			
Overall BC[†]	98.8 [96.7-99.6]	94.3 [91.3-96.3]	96.5 [94.7-97.7]
High-grade disease [a]	100 [98.6-100]	98.5 [96.2-99.4]	99.2 [98.0- 99.7]
**Rule-out rate**, % [95% CI]	76.6 [72.2-80.5]	62.0 [57.4-66.5]	68.4 [65.0-71.6]

[a] High-grade disease indicates Ta-HG, CIS, T1, T2, and T3.

Abbreviation: CI, confidence interval; NPV, negative-predictive value; PPV, positive-predictive value

Supplementary Table S7. Pre-test and post-test probabilities of detecting BC in the pooled cohort with microhematuria

Pre-test probability	Positive likelihood ratio (LR+)[b]	Post-test probability[d] of BC Based on LR+	Negative likelihood ratio (LR-)[c]	Post-test probability[d] of BC Based on LR-
0.5%[a]	5.0	2.5%	0.1	0.05%
1.0%[a]	5.0	4.8%	0.1	0.1%
2.0%[a]	5.0	9.3%	0.1	0.2%
3.0%[a]	5.0	13.0%	0.1	0.31%
8.0% (Dataset incidence)	5.0	30.3%	0.1	0.86%

[a] Pre-test probability scenario (assumed values). Dataset incidence reflects the actual prevalence observed in the pooled cohort.

[b] Positive likelihood ratio (LR+) was calculated as the true positive rate (sensitivity) divided by the false positive rate (1–specificity), representing the positive likelihood ratio (LR+)

[c] Negative likelihood ratio was calculated as the false negative rate (1–sensitivity) divided by the true negative rate (specificity), representing the negative likelihood ratio (LR–)

[d] Post-test probability represents the updated likelihood of bladder cancer after testing, obtained by applying the likelihood ratio to the pre-test probability (based on prevalence or risk group).

Supplementary Table S8. Performance comparison of BCD Plus, Cytology, and NMP22

**Parameter**	**BCD Plus (n = 784)**	**Cytology (n = 784)**	**p-value***
**Sensitivity,** % [95% CI]			
Overall BC	87.3 [80.8-92.1]	32.9 [25.4-41.1]	<0.001
High-grade disease [a]	96.4 [91.1-99.0]	42.9 [33.6-52.6]	<0.001
**Specificity,** % [95% CI]	81.3 [78.0-84.2]	99.7 [98.9- 99.9]	<0.001

**Parameter**	**BCD Plus (n = 718)**	**NMP22 (n = 718)**	**p-value***
**Sensitivity,** % [95% CI]			
Overall BC	88.4 [82.0-93.1]	43.2 [35.0-51.6]	<0.001
High-grade disease [a]	97.3 [92.2-99.4]	53.6 [43.9-63.2]	<0.001
**Specificity,** % [95% CI]	80.2 [76.7-83.4]	92.8 [90.4-94.8]	<0.001

[a]High-grade disease indicates Ta-HG, CIS, T1, T2, and T3.

[*]*P* values were calculated using McNemar's test for sensitivity and specificity.

Supplementary references

[1] Unal I. Defining an Optimal Cut-Point Value in ROC Analysis: An Alternative Approach. *Comput Math Methods Med*. 2017:3762651.
